# Evaluating urinary estrogen and progesterone metabolites using dried filter paper samples and gas chromatography with tandem mass spectrometry (GC–MS/MS)

**DOI:** 10.1186/s13065-019-0539-1

**Published:** 2019-02-04

**Authors:** Mark Newman, Suzanne M. Pratt, Desmond A. Curran, Frank Z. Stanczyk

**Affiliations:** 1 Precision Analytical Inc., 3138 NE Rivergate Street #301C, McMinnville, OR 97128 USA; 2 Suzanne Pratt Works LLC, 7114 SE 18th Avenue, Portland, OR 97202 USA; 3 Department of Obstetrics and Gynecology, and Preventive Medicine, University of Southern California, Keck School of Medicine, Los Angeles, CA USA

**Keywords:** Dried filter paper, DUTCH, Estradiol, GC–MS/MS, Hormone replacement therapy, Pregnanediol, Progesterone, Reproductive hormones, Subfertility

## Abstract

**Background:**

Measuring concentrations of metabolites of estradiol and progesterone in urine, instead of measuring serum concentrations, is common in research and also is used in patient care. The primary aim of this study was to demonstrate that analysis of urine samples dried on filter paper by gas chromatography with tandem mass spectrometry (GC–MS/MS) provides results similar to serum analyzed by radioimmunoassay (RIA). Secondary aims were to show that collection of four samples during the day (4-spot method) can be substituted for a 24-h collection, and that analysis of urine from dried samples is equivalent to liquid urine samples.

**Methods:**

This prospective observational study compared results of urine and serum analyses. Urine samples from women throughout the menstrual cycle and single samples from postmenopausal women were evaluated. Urine was collected onto filter paper and dried. Dried urine was extracted, hydrolyzed, and derivatized prior to analysis by GC–MS/MS. Hormone concentrations were normalized to creatinine. Single samples were used to compare results of 24-h urine collection to the 4-spot method from a separate population of women and men. A subset of these samples were used to compare results from dried urine to liquid urine.

**Results:**

The primary study showed good reliability in the comparisons between the dried urine and serum assays. During the menstrual cycles of a subset of four women, urine metabolite concentrations followed the same pattern as serum concentrations. Comparison of 4-spot to 24-h urine collections and of dried to liquid urine measurements had intraclass correlation coefficients (ICC) greater than 0.95, indicating excellent agreement.

**Conclusions:**

For estradiol and progesterone, the dried urine assay is a good surrogate for serum testing. The 4-spot method can be used instead of 24-h urine collections and dried urine results are comparable to liquid urine. The dried urine assay is useful for some clinical assessments of hormone disorders and may be useful in large epidemiologic studies due to ease of sample handling.

**Electronic supplementary material:**

The online version of this article (10.1186/s13065-019-0539-1) contains supplementary material, which is available to authorized users.

## Background

Analysis of reproductive hormones is commonly performed in epidemiological studies, clinical research, and patient care. Estrogen and progesterone measured in serum and plasma or their metabolites measured in urine provide similar information about ovarian steroid production [1, 2]. To evaluate diurnal, circadian, or monthly variations in hormone levels, frequent sampling may be needed and patients can easily collect the urine samples. For studies analyzing the monthly pattern of reproductive hormones (menstrual cycle mapping), evaluation of daily hormone levels from the first urine of the day is usually sufficient [3]. However, to evaluate hormones that may have a circadian rhythm that is either biological or due to hormone replacement therapy, a representation of the entire day may be beneficial. To provide an alternative and possibly more convenient collection strategy, we compare the results of urine collected at 4 times during the day (4-spot method) with a complete 24-h collection.

We also compare results of dried urine samples to liquid urine, as an additional potential convenience for patients. The basic method for collection of urine samples onto filter paper was developed decades ago to facilitate sample acquisition in screening newborn/metabolic disorders and has been adapted [4]. Dried filter paper samples provide convenience of collection and ease of transport. It has also been suggested that using dried urine samples could increase participation rates in some studies, particularly in regions that do not have reliable access to refrigeration [5].

Recent advances in mass spectrometry technology have enabled its use for routine analysis of steroid hormones in clinical and research laboratories [6–8]. Our diagnostic laboratory has developed methods for analyzing steroid hormone metabolites from small volumes of dried urine using gas chromatography with tandem mass spectrometry (GC–MS/MS). The methodology provides accurate and precise quantification at the lower concentrations of urinary estrogen and progesterone metabolites found in postmenopausal women, children, and men. A complete evaluation of all of the urinary metabolites is obtained using GC–MS/MS that provides for high resolution of very closely related structures (isomers) [6]. All of conjugated forms of estradiol and progesterone metabolites are cleaved back to the parent hormone and measured directly.

The primary aim of the study is to demonstrate that measuring urinary metabolites of estradiol and progesterone from dried filter paper samples analyzed by GC–MS/MS provide results comparable to measuring serum estradiol and progesterone with standard RIA methodology in premenopausal and postmenopausal women. A secondary aim is to show that collection of dried urine samples at 4 time points over a 10 to 14 h period (4-spot method) provides results comparable to a complete 24-h urine collection. Additionally, we show that results obtained using dried urine extracted from filter paper are equivalent to those obtained for liquid urine.

## Materials and methods

A prospective observational study was carried out between February and November of 2015. Previously collected and stored blood and urine samples from healthy adults were analyzed. Informed consent was obtained prior to the study and all individual data was de-identified, in compliance with the Helsinki Declaration. Serum analyses for estradiol and progesterone were performed by Dr. Stanczyk’s laboratory at the University of Southern California, Los Angeles, CA, USA. Urine analyses for estradiol (E_2_), estrone (E_1_), 5α-pregnane-3α, 20α-diol (5α-pregnanediol, αPg) and 5β-pregnane-3α, 20α-diol (5β-pregnanediol, βPg) were conducted at Precision Analytical, Inc., McMinnville, OR, USA.

The study used previously collected, de-identified samples and it was determined by the National University of Natural Medicine Institutional Review Board to meet the criteria for exemption status (IRB number: MN020918).

### Study populations and sample collection

For the primary study, comparing results of serum to urine analyses, samples from four premenopausal and eight postmenopausal women were used. Multiple samples of blood and urine from the premenopausal women had been collected on various days throughout their menstrual cycle (total n = 44; range: 8 to 13 samples per individual). Single samples of blood and urine from the postmenopausal women were analyzed, as there was no cyclical pattern to monitor. Inclusion of postmenopausal women provided data for the lower end of the measured range of the hormones. Women were excluded from the study if they had used hormonal contraception or hormone replacement therapy within 1 year of testing or were pregnant. Blood was collected (2 mL) by capillary finger stick; serum (1 mL) was separated then frozen at − 80 °C, and shipped overnight on ice. Urine (approximately 2 mL) from the first urine of the day was collected onto filter paper, dried, frozen, and later transported at room temperature to the lab where the samples were stored at − 80 °C until analysis.

For the secondary studies, a group of 26 individuals was used to compare samples from the 4-spot method to 24-h urine collection. A subset of the group (18 individuals) was used for the dry to liquid urine comparison. Individuals were not excluded based on current or recent hormone therapies. These study populations included both males and non-pregnant females to provide for a larger sample size and a range of expected values for hormones not included in this report (such as testosterone) as the goal was to compare measurement values for differing methodologies. Urine from 24-h collections was delivered to the laboratory, the total volume was measured, and an aliquot was frozen and stored at -80 °C until tested. Dried samples were also stored at − 80 °C until analyzed.

Urine samples were collected by saturating 2 × 3 inches of filter paper (Whatman Body Fluid Collection Paper or equivalent) with urine. Instructions were given to completely saturate the paper, which was usually easily accomplished. The paper was left exposed at room temperature for 24 h to dry. The 4-spot method used samples collected at four times during the day that spanned 10–14 h of the 24-h period. Urine collections were taken from the first urine of the day, 2 h later, at dinnertime, and before bed. The first morning collection captures the overnight period of 6–8 h with each of the other three collections accounting for approximately 2 h of the day (up to 6 h total). During the same day, liquid urine samples for the 24-h collection were added to a container with approximately 1 g of boric acid. The four dried urine samples removed a total of about 8 mL of urine from the 24-h collection. This was considered negligible and was not accounted for.

### Serum hormone analysis

Estradiol, was quantified in serum by a previously described RIA method [9, 10]. Prior to the RIA, steroids were extracted with hexane:ethyl acetate (3:2) and estradiol was separated by Celite column partition chromatography, using 40% ethyl acetate in isooctane. High specific activity tritiated estradiol (500 dpm) was added to each serum sample before the extraction step in order to follow and correct for procedural losses. Inter-assay coefficients of variation for quality control samples in the estradiol RIA ranged from 9.0% to 12.0%. The sensitivity of the estradiol RIA is 2 pg/mL.

A commercial RIA kit (Cisbio Bioassays, Codolet, France), using a highly specific antibody, was used to measure progesterone. The assay was carried out in progesterone antibody-coated tubes in conjunction with an iodinated progesterone derivative. After a 2-h= incubation at 37 °C, the contents of the tubes were aspirated and the tubes were washed. The inter-assay coefficients of variation were < 10% for the range of progesterone from 0.12 to 36 ng/mL. The progesterone RIA assay sensitivity is 0.12 ng/mL.

### Urine hormone metabolite analysis

The urinary metabolites were analyzed using proprietary in-house assays referred to as Dried Urine Testing for Comprehensive Hormones (DUTCH) on the Agilent 7890/7000B GC–MS/MS (Agilent Technologies, Santa Clara, CA, USA). The equivalent of approximately 600 μl of urine was extracted from the filter paper using 2 mL of 100 mM ammonium acetate adjusted to a pH of 5.9. Aliquots of the conjugated hormones were transferred to a C18 solid phase extraction (SPE) column (UCT LLC, Briston, PA, USA), eluted using methanol, and the eluate was dried under nitrogen at 40 °C. The conjugated hormones were then hydrolyzed from their glucuronide and sulfate forms to free forms using enzymes from Helix pomatia (Sigma-Aldrich, St. Louis, MO, USA) in acetate buffer (55 °C, 90 min). The enzymatic reaction was quenched with sodium hydroxide and the hormones extracted with ethyl acetate. The ethyl acetate extracts were dried under nitrogen at 40 °C. The analytes were derivatized using a mixture of 100 μL acetonitrile and 50 μL bis(trimethylsilyl)trifluoroacetamide (Sigma-Aldrich, St. Louis, MO, USA) for 30 min at 70 °C. Internal standards (estradiol-D5, Steraloids, Newport, RI, USA) were added prior to ethyl acetate extraction, and the percentage recovery after all assays was > 90%. Derivatized extract (1.6 μL) was injected into the GC–MS/MS. Samples were analyzed along with a standard curve spanning the expected range of concentrations along with a series of controls. Instrument conditions for the oven were an initial temperature of 130 °C increasing to 200 °C at 25 °C/min, then to 230 °C at 4.3 °C/min, and finally to 290 °C at 25 °C/min. Multiple reaction monitoring transitions were 416 > 285 for E_2_, 342 > 257 for E_1_, 269 > 187 for αPg, and 449 > 103 for βPg. Creatinine was measured using a conventional colorimetric (Jaffe) method, after initial extraction from the filter paper. The average inter-assay coefficients of variation were 8% for E_2_, 10% for E_1_, 12% for αPg, and 13% for βPg. Sensitivities of the assays were as follows: E_2_, E_1_, and αPg, 0.2 ng/mL; βPg, 10 ng/mL.

Urine reference ranges were determined by testing a prior cohort of 600 premenopausal women. Urine hormone metabolite concentrations were normalized to creatinine to account for variations in urine concentration and to compensate for any variations in filter paper saturation during collection. The luteal reference range used is the 20th to the 80th percentile for this population.

### Dried urine sample stability

A stability study was conducted on urine from four individuals. Aliquots were applied to filter paper and dried. These were stored at room temperature and then frozen at − 80 °C at 15 different time points ranging from day 0 to day 84 (n = 60). Finally, all samples were tested in a single batch to assess for reproducibility of the measurements, and thus, stability of the four analytes at room temperature for up to 84 days.

### Statistical methods

The post hoc power calculation based on the primary study with a sample size of 12 subjects provided more than 85% power to detect an interclass correlation coefficient of at least 0.75 between serum and urine values, before accounting for repeated measures. The use of repeated measures in the four premenopausal individuals increased the power to greater than 90% to detect a Spearman correlation of at least 0.6. Alpha was set at 0.05, and all analyses generated 2-sided p-values. The statistical analyses were performed using SAS/STAT^®^ software, Version 9.3 (SAS Institute Inc., Cary, NC, USA).

Hormonal measures are expressed as median (interquartile range (IQR)), as they were not normally distributed. Similarly, because the data were not normally distributed, only non-parametric tests were used in the analyses. Spearman correlation coefficients were used to determine interclass associations between variables. Wilcoxon and Mann–Whitney tests were used to assess differences between men and women. Reference ranges were employed to standardize the serum and urine values for direct comparison, using min–max scaling, i.e. (observed value − minimum reference)/(maximum reference − minimum reference). Consistency and agreement between the standardized serum and urine measures were assessed using intra-class correlation coefficients (ICC). ICCs differ from the more familiar interclass correlations (e.g., Pearson and Spearman correlation coefficients) by assessing the agreement of a measure between groups [11]. The ICC ranges from 0 to 1; the higher the value, the more closely the two measures are in perfect agreement. Mixed models to account for repeated measures and using a variance components covariance structure were used to assess whether observed differences between serum and urine standardized values were significant, and to determine differences between premenopausal and postmenopausal women. Consistency of 4-spot versus 24-h urine collections and dried versus liquid urine samples were evaluated with ICCs, while comparisons of differences between measures within an individual were assessed using signed rank tests. Because the hypotheses of this paper were intrinsically correlated and each question was of independent interest, no adjustments were made for multiple comparisons.

## Results

### Study populations

The primary study evaluated 52 samples: 44 premenopausal from day four to the end of the menstrual cycles and 8 postmenopausal. The 4-spot to 24-h comparison had one sample each from 26 individuals, and a subset of these (n = 18) used for the liquid to dry comparison had one sample each from 18 individuals. Characteristics of the populations and descriptive statistics are provided in Table 1.

**Table 1:** Study population characteristics and hormonal concentrations

Variable	Primary study: serum versus dried urine^c^			Secondary studies^d^		
	All	Premenopausal	Postmenopausal	All	Female	Male
Age (mean ± SD)	50.1 ± 19.2	24.8 ± 5.6	62.8 ± 3.9	36.8 ± 14.5	33.7 ± 7.8	37.8 ± 18
Group size	N = 12	N = 4	N = 8	N = 26	N = 15	N = 11
Race	11 Caucasian 1 Hispanic	3 Caucasian 1 Hispanic	Caucasian	Caucasian	Caucasian	Caucasian
Serum Estradiol (pg/ml)	15.38 (7.84, 91.90)	129.21 (103.00, 147.53)	9.37 (6.69, 15.38)^a^	N/A	N/A	N/A
Urinary Estradiol (ng/mg-Cr)	0.39 (0.11, 1.87)	3.02 (2.19, 3.72)	0.18 (0.04, 0.39)^b^	1.68 (0.92, 3.81)	3.34 (0.85, 4.92) ^b^	1.22 (0.92, 1.64)
Urinary Estrone (ng/mg-Cr)	7.5 (2.7, 13.4)	17.0 (14.5, 17.7)	3.8 (1.7, 7.5)^a^	14.4 (7.1, 18.8)	18.6 (6.7, 25.4)	9.2 (7.1, 14.4)
Serum Progesterone (ng/ml)	0.24 (0.08, 1.57)	2.79 (1.54, 5.15)	0.12 (0.07, 0.54)	N/A	N/A	N/A
Urinary αPg (ng/mg-Cr)	21.3 (13.5, 49.5)	77.1 (53.1, 142.5)	14.1 (12.8, 26.1)	60.9 (29.7, 242.0)	133.3 (18.8, 329.5)	51.4 (29.7, 93.1)
Urinary βPg (ng/mg-Cr)	97.9 (58.5, 174.0)	282.6 (197.9, 445.3)	80.0 (41.3, 97.9)	204.3 (113.5, 466.6)	205.8 (113.5, 1733.1)	197.8 (80.3, 354.4)

Hormone data presented as median (IQR). Urine values are from the dried urine assay

Cr, creatinine; αPg, 5α-pregnanediol; βPg: 5β-pregnanediol

^a^p < 0.01

^b^p < 0.05 for differences between groups (pre- versus post-menopausal women or males versus females), as assessed with mixed models for the primary study and Wilcoxon and Mann–Whitney tests for the secondary studies

^c^All calculations of hormonal measures account for repeated measures within the premenopausal individuals using mixed models

^d^Of the 26 individuals (15 females, 11 males) who participated in the comparison of the 4-spot versus 24-h urine assays, 18 individuals (10 females, 8 males) were used to assess comparability of dried versus liquid urine samples

### Stability of dried urine samples

The four hormone metabolites showed good stability when stored at room temperature for almost 3 months. The average coefficient of variation between the first and last measurement was 4.9 ± 2.4% for E_2_, 7.1 ± 3.5% for E_1_, 4.1 ± 2.9% for αPg, and 7.3 ± 3.2% for βPg. After 84 days, the measures of E_2_, E_1_, and αPg were unchanged with nonsignificant differences of 2.6 ± 8.1% (p = 0.57), 4.3 ± 11.5% (p = 0.51), and 5.8 ± 4.3% (p = 0.08), respectively. There was a slight increase in βPg by 10.3 ± 4.5% (p = 0.02).

### Primary study: Dried urine compared to serum

**Fig. 1:**
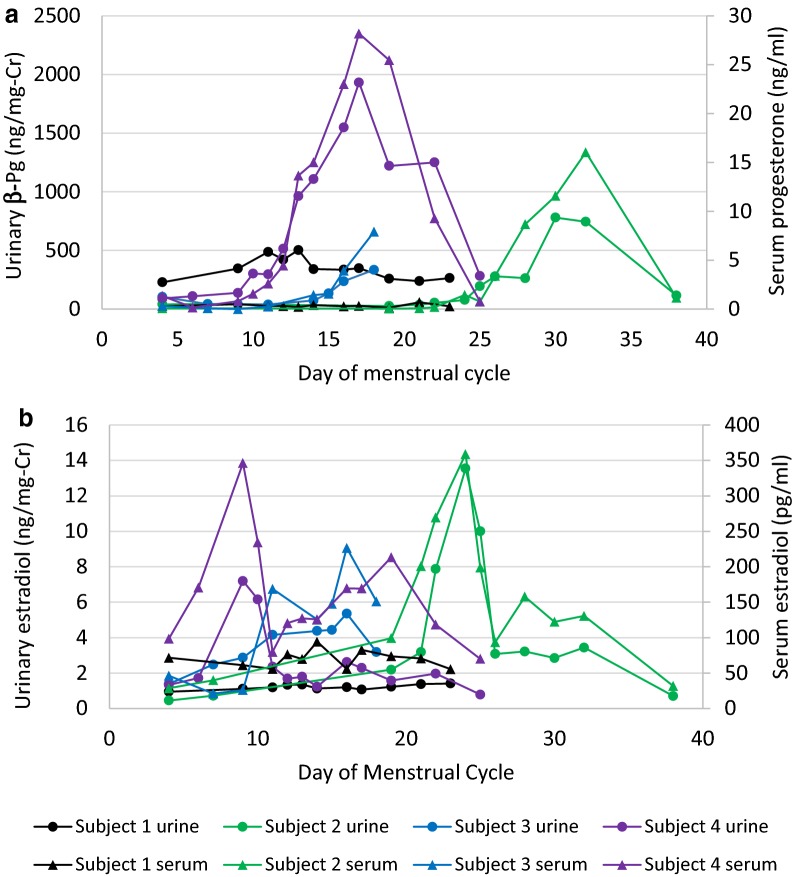
Hormone profiles of serum progesterone versus urinary 5β-pregnanediol (**a**) and serum estradiol and urinary estradiol (**b**) in four premenopausal women. Cr, creatinine; βPg, β-pregnanediol

**Fig. 2:**
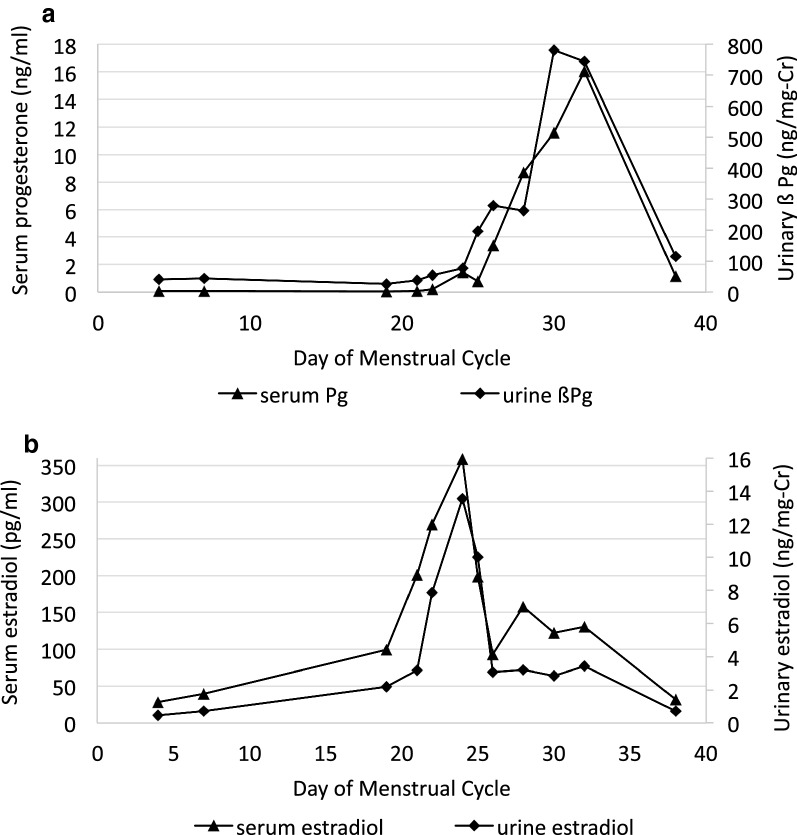
Hormone profiles of serum progesterone versus urinary β-pregnanediol (**a**) and serum versus urinary estradiol (**b**) in one premenopausal woman’s cycle. Metabolites of subject 2. Cr, creatinine; βPg, β-pregnanediol

The primary study evaluated whether reproductive hormone metabolites, as measured in a dried urine sample by GC–MS/MS, provide information comparable to serum hormone concentrations measured by a conventional RIA method. Assessment of consistency between standardized measurements from serum to dried urine assays using ICCs revealed substantial reliability between the assays in all comparisons (Table 2) [12]. There were no significant differences between the urine and serum assays for E_2_, E_1_, and αPg. However, standardized βPg was consistently greater than standardized serum progesterone within individuals indicating a small, but systematic difference (p = 0.03). All four urinary hormone metabolites followed the pattern of results obtained from serum with similar timing of peaks and troughs. The cycle maps for urinary βPg and estradiol are shown in Figs. 1, 2 and urinary αPg and estrone in Additional file 1. Subjects 2, 3, and 4 showed the usual luteal surge of progesterone, whereas subject 1 is presumed to be anovulatory with no surge in either of the progesterone metabolites. The individual data for the 4 premenopausal women are found in Table 3.

**Table 2:** Comparison of urinary hormone metabolites measured with the dried urine assay to serum hormones concentrations measured by RIA

	Urinary estradiol (ng/mg-Cr) v serum estradiol (pg/ml)	Urinary estrone (ng/mg-Cr) v serum estradiol (pg/ml)	Urinary αPg (ng/mg-Cr) v serum progesterone (ng/ml)	Urinary βPg (ng/mg-Cr) v serum progesterone (ng/ml)
Dried urine assay	0.39 (0.11, 1.87)	7.47 (2.67, 13.44)	21.34 (13.54, 49.48)	97.92 (58.47, 174.01)
Serum assay	15.38 (7.84, 91.90)	15.38 (7.84, 91.90)	0.24 (0.08, 1.57)	0.24 (0.08, 1.57)
Standardized urine measures	− 0.05 (− 0.32, 0.46)	0.29 (− 0.26, 0.57)	0.89 (− 0.01, 0.34)	0.09 (− 0.20, 0.46)
Standardized serum measures	0.33 (0.22, 0.41)	0.33 (0.22, 0.41)	0.07 (− 0.05, 0.30)	0.07 (− 0.05, 0.30)
p-value for paired comparisons of standardized measures	0.47	0.99	0.32	0.03
ICC	0.78	0.83	0.83	0.85

Data presented as median (IQR). Standardization of the urine and serum measures was accomplished using the min–max scaling with established reference ranges. The p-values for the paired comparisons were obtained using mixed models to account for repeated measures

ICC, intraclass correlation coefficient; Cr, creatinine

**Table 3:** Individual data for urinary and serum hormone levels in 4 premenopausal women

	Day of cycle	Urinary 5α-pregnanediol (ng/mg Cr)	Urinary 5β-pregnanediol (ng/mg-Cr)	Urinary estrone (ng/mg-Cr)	Urinary estradiol (ng/mg-Cr)	Serum progesterone (ng/ml)	Serum estradiol (pg/ml)
Subject 1	4	49.32	229.20	7.55	0.95	0.29	71.54
	9	62.81	346.42	7.63	1.11	0.51	61.00
	11	67.65	487.85	8.62	1.20	0.31	55.78
	12	90.06	422.07	12.05	1.34	0.31	76.28
	13	86.91	503.89	10.48	1.35	0.22	69.76
	14	60.75	340.59	8.34	1.12	0.41	94.11
	16	47.38	336.25	8.22	1.20	0.29	55.80
	17	51.39	348.51	8.66	1.07	0.34	82.70
	19	52.62	258.78	8.22	1.23	0.14	73.66
	21	58.27	239.14	9.50	1.38	0.70	70.59
	23	62.93	265.09	10.06	1.41	0.29	55.44
Subject 2	4	15.81	41.04	3.03	0.45	0.08	28.52
	7	20.85	43.55	3.82	0.72	0.08	39.88
	19	21.72	26.57	8.08	2.19	0.06	99.32
	21	17.36	37.83	9.29	3.18	0.09	200.95
	22	24.14	54.48	27.73	7.87	0.20	269.52
	24	25.30	77.84	46.24	13.55	1.44	358.85
	25	63.19	196.32	38.49	10.01	0.78	198.80
	26	104.10	279.14	17.84	3.08	3.41	93.36
	28	136.19	263.40	11.88	3.21	8.69	157.82
	30	334.19	780.19	12.77	2.85	11.58	122.30
	32	289.28	744.82	13.59	3.44	16.04	130.67
	38	44.87	115.68	2.54	0.71	1.15	31.86
Subject 3	4	11.83	105.41	7.17	1.39	0.32	46.29
	7	9.63	42.28	12.43	2.48	0.08	21.09
	9	8.34	41.79	12.68	2.87	0.00	26.05
	11	9.91	39.64	16.90	4.16	0.26	168.71
	14	16.67	73.06	22.91	4.38	1.44	125.45
	15	26.21	134.70	20.01	4.44	1.57	147.69
	16	51.81	238.10	22.07	5.36	3.92	226.53
	18	59.46	335.32	27.28	3.19	7.92	151.00
Subject 4	4	44.36	91.39	9.57	1.33	1.34	98.25
	6	42.10	109.85	13.35	1.72	0.17	170.76
	9	53.99	138.99	37.93	7.20	0.80	346.16
	10	118.56	303.51	39.86	6.16	1.57	234.24
	11	122.49	296.39	21.14	2.36	2.60	79.68
	12	196.42	514.70	14.29	1.69	4.45	120.46
	13	384.23	964.09	14.11	1.79	13.66	127.17
	14	460.59	1108.31	12.71	1.23	15.01	125.75
	16	589.30	1548.58	20.19	2.62	23.01	169.62
	17	691.31	1931.82	15.36	2.29	28.17	169.17
	19	566.98	1220.46	11.39	1.57	25.46	213.47
	22	463.24	1250.27	14.76	1.97	9.29	118.36
	25	110.89	283.56	5.09	0.78	0.78	70.13

### Secondary studies

#### 4-spot versus 24-h collection

**Fig. 3:**
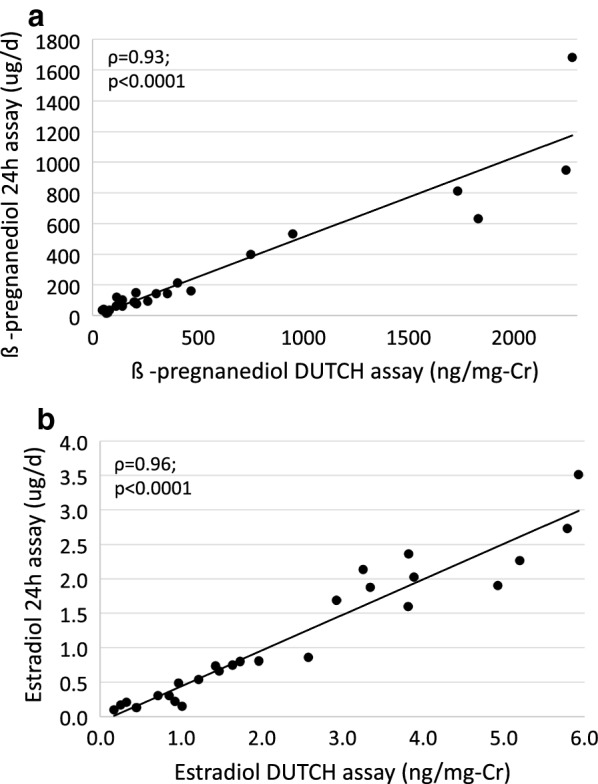
Interclass correlations of 24-h urine collections for β-pregnanediol (**a**), estradiol (**b**), versus the 4-spot assay. Correlation coefficients reported are Spearman correlations. Cr, creatinine; βPg, 5β-pregnanediol

The measurement of hormone metabolites in urine samples collected four times throughout the day (4-spot) covering an average of 12 h (range 10-14 h) are comparable to the *gold standard* of a 24-h urine collection (Table 4). The ICC was greater than 0.95 for all comparisons, indicating almost perfect agreement between the 4-spot collection technique and the gold standard assay. Despite the excellent agreement, the 4-spot results were slightly, but consistently higher than the 24-h urine results for the urinary E_1_, and E_2_ concentrations in the paired comparisons with the 24-h urine collection (p < 0.05 for both). Spearman correlation analysis with 24-h urine data expressed as ug/day versus 4-spot assay results showed excellent correlation (Fig. 3 and Additional file 2).

**Table 4:** Comparison of the 4-spot method with a 24-h urine collection (n = 26)

Variable	24 h urine collection (μg/day)	24 h urine collection (ng/mg-Cr)	4-spot (ng/mg-Cr)	Difference [95% confidence interval]	ICC
Estradiol	0.77 (0.30, 1.90)	1.73 (0.80, 3.26)	1.68 (0.92, 3.81)	0.16 [0.01, 0.31]*	0.97
Estrone	6.0 (3.3, 9.9)	13.7 (6.3, 19.2)	14.4 (7.1, 18.8)	0.8 [0.1, 1.4]^*^	0.98
5α-pregnanediol	34.9 (13.6, 88.4)	71.4 (28.9, 257.2)	60.9 (29.7, 242.0)	− 11.6 [− 38.7, 15.6]	0.97
5β-pregnanediol	99.2 (60.7, 214.0)	243.9 (99.2, 408.9)	204.3 (113.5, 466.6)	44.0 [− 27.2, 115.2]	0.96

Data presented as median (IQR)

Differences were calculated using a signed rank test (4-spot—24 h assay), as variables were not normally distributed. Note that ICCs were calculated between measurements expressed in identical units. Results for the 24-h collection are reported as ng/mg-Cr for ICC calculation and also as μg/day, the usual units for reporting results of 24-h collections

*Cr* creatinine, *ICC* intraclass correlation coefficient

* Estrone p = 0.03, estradiol p = 0.0499

#### Liquid versus dry urine samples

**Fig. 4:**
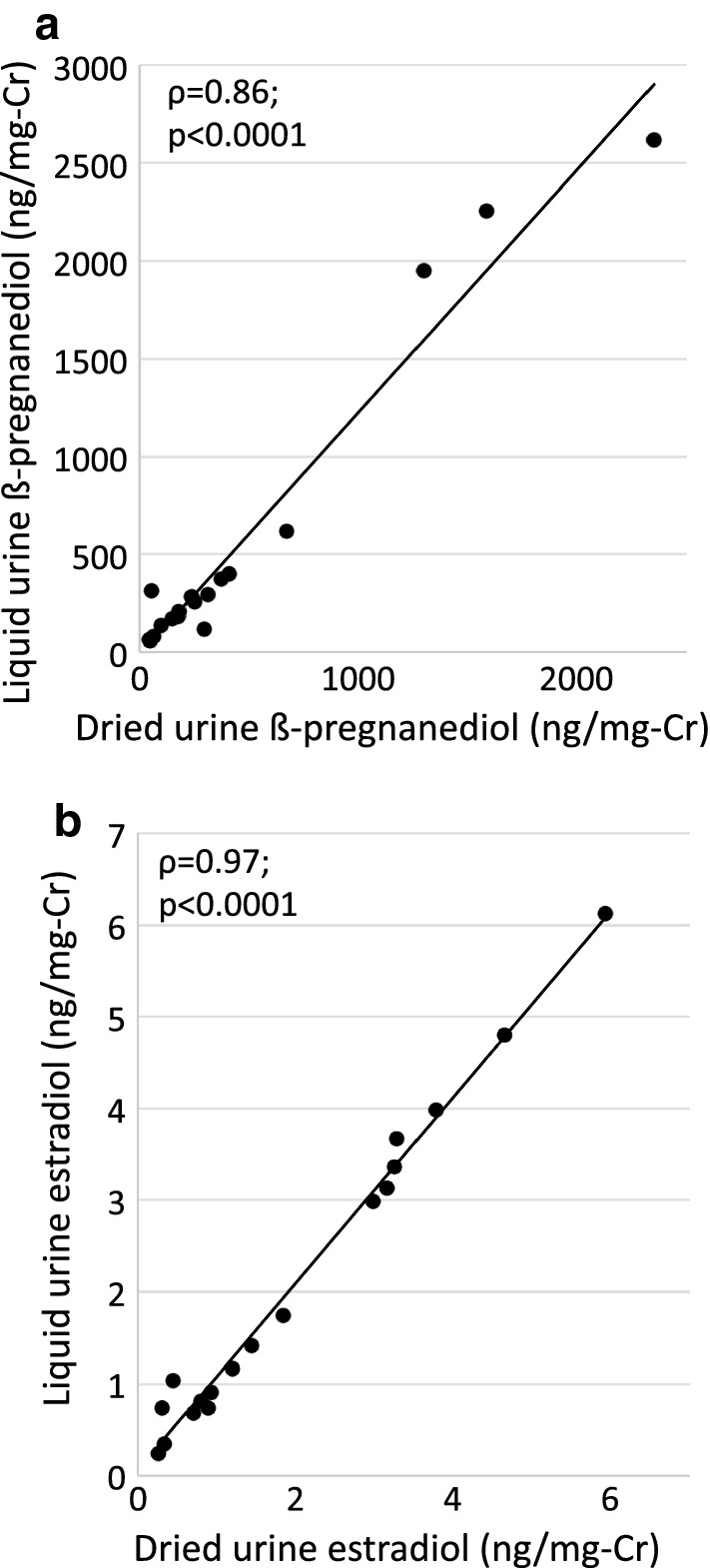
Interclass correlations of dried versus liquid urine for β-pregnanediol (**a**) and estradiol (**b**). Correlation coefficients reported are Spearman correlations. Cr, creatinine; βPg, β-pregnanediol

There was no difference in analyzing liquid urine or dried urine samples extracted from filter paper. Results from a dried versus a liquid urine sample showed excellent consistency, as assessed by ICCs (Table 5**)**. Despite the excellent agreement, the dried urine assay measurement was consistently lower than the liquid assay for urinary E_1_ and βPg concentrations in the paired comparisons (p = 0.04 for both). Spearman correlation for the 4 urinary metabolites was excellent (Fig. 4 and Additional file 3).

**Table 5:** Comparison of dried versus liquid urine analysis (n = 18)

Variable	Dried	Liquid	Difference [95% confidence interval]	ICC
Estradiol (ng/mg-Cr)	1.32 (0.70, 3.26)	1.29 (0.74, 3.36)	− 0.09 [− 0.19, 0.01]	0.99
Estrone (ng/mg-Cr)	10.78 (6.26, 18.73)	12.78 (7.22, 20.65)	− 1.28 [− 2.47, − 0.09]^*^	0.96
5α-pregnanediol (ng/mg-Cr)	65.81 (27.49, 257.23)	62.29 (32.18, 248.25)	− 5.72 [− 29.16, 17.71]	0.98
5β-pregnanediol (ng/mg-Cr)	243.90 (99.22, 408.88)	270.25 (135.96, 400.42)	− 98.67 [− 211.46, 14.13]*	0.94

Data presented as median (IQR)

Differences were calculated using a signed rank test (dried-liquid assay), as variables were not normally distributed

*ICC* intraclass correlation coefficient

* p = 0.04

## Discussion

We have evaluated the performance of a validated proprietary assay for estradiol and progesterone metabolites using dried urine samples and GC–MS/MS analysis in comparison with results of serum analysis using conventional RIAs.

Our primary study showed that the measurement of urinary hormone metabolites in the dried urine assay provides menstrual cycle maps comparable to those derived from serum hormone levels in individual premenopausal women (Figs. 1, 2 and Additional file 1). The urine results showed substantial agreement with serum results based on intraclass correlations for the group of premenopausal and postmenopausal women for all metabolites supporting use of the urinary assay as a substitute for serum analysis. The urinary results were not significantly different than serum when analyzed using standardized reference ranges, except for βPg. Despite the statistical difference for βPg it is unlikely to be clinically significant. The βPg levels may be consistently higher than those of serum progesterone due to a number of factors that include variations in protein binding in serum, cross-reactivity in the RIA, and differences in that populations of premenopausal women used to develop the reference ranges. Menstrual cycle maps of four premenopausal women were compared for serum and urine measures. Although the sample size was small, because there were multiple samples from each individual over the course of their menstrual cycle, the repeated measures analysis provided good statistical power and confidence in the comparisons.

We also showed that using a 4-spot collection technique is equivalent to doing a complete 24-h urine collection as evaluated by intraclass correlations. Although the 4-spot method consistently overestimated the estrogen metabolites compared to the 24-h urine collection, both differences were less than 10% of the total and not likely to be clinically relevant. Factors that may contribute to these small differences include individual variation, phase of the menstrual cycle, and timing of the 4-spot collections. With the excellent agreement demonstrated by the intraclass correlation coefficients, the 4-spot collection method may be useful for evaluating some postmenopausal women considering HRT. For monitoring the effectiveness of some HRT, such as transdermal estrogen that avoids first-pass metabolism, evaluation of urinary hormone metabolites may be useful. Routes of administration, such as oral or sublingual, undergo first-pass hepatic metabolism and urine evaluation may not be appropriate.

The final part of our study established that using urine dried onto filter paper provides almost identical results to testing liquid urine. This result was expected, but we do not believe this information has been published previously. While the collection method of urine onto filter paper is easy, it is not infallible, and occasionally the paper may not become completely saturated. For this reason, the urinary hormone metabolites are indexed to creatinine concentration in our studies to account for possible variation in saturation of the filter paper.

Use of a dried filter paper sample provides a small volume of urine that is sufficient for GC–MS/MS analysis, and limited repeat testing. If more volume is needed, additional samples can be collected, either at the time of initial collection or at a later date. Dried samples require extraction from the paper prior to analysis, an additional step as compared to using liquid urine, but in the commercial laboratory setting, workflows are optimized and throughput efficient. Using filter paper samples provides for ease of storage and transportation and may facilitate studies in a broader range of world settings, as neither refrigeration nor freezing are required. Sample stability will vary depending on the compounds of interest, but our samples had good stability at ambient temperatures for nearly 3 months.

Assessments of urinary hormone metabolites can be used in place of serum hormone analysis in some situations, but there are important variables to understand. Timing of sample acquisition is important, as serum testing provides information about status at the moment of collection, but urinary testing provides information from a span of time and with a lag due to metabolic processing. For hormones with pulsatile secretion such as progesterone [13] (serum concentrations may vary fivefold or more from minute to minute during luteal phase), the use of urine samples mitigates the variability of serum testing and provides a result that represents 6–8 h of time (for a waking sample).

Metabolism of steroid hormones produces a number of conjugates in urine and the proportion of each can vary between individuals. For instance, metabolites of E1 and E2 include glucuronide and sulfate forms of the hormones. Pregnanediol may exist as a glucuronide at the 3 position, at the 20 position or at both positions and also exists as both αPg and βPg. Different individuals may have significantly different distributions of metabolites and their conjugates. With the GC–MS/MS analysis, all of the conjugates are cleaved and measured providing for a potentially more complete representation of the hormone profile. In addition to individual variation, testing urine metabolites assumes that the individuals have normally functioning phase II metabolism of estradiol and estrone as well as the phase I metabolites of progesterone. Although we are not aware of any defects in estrogen or progesterone metabolism, there are known defects of phase II metabolism of testosterone that may lead to falsely low results, and the possibility cannot be ruled out [14].

The use of GC–MS/MS confers some advantages over other analytical tools. Laboratories often choose between GC and MS and liquid chromatography with tandem mass spectrometry (LC–MS/MS) assays for urinary evaluations [6–8]. GC–MS offers better chromatographic separation of similar compounds (such as isomers), but assays may suffer from a lack of sensitivity and selectivity. The LC–MS/MS combination systems offer a high degree of accuracy and sensitivity needed for small sample volumes and for many polar compounds allows for testing without the need for derivatization. Throughput is often increased, but the chromatographic separation is less than GC can provide. Increased chromatographic separation is helpful in cases where the signal from the mass spectrometer is not unique for two compounds. For example, isobaric estrogen metabolites may not give a unique signal on the GC–MS/MS and thus would have to be separated chromatographically.

In this study, we evaluated only urinary metabolites of estradiol and progesterone, although the DUTCH method is designed to also measure androgen metabolites, cortisol metabolites, free cortisol, and melatonin. The 4-spot collections are helpful for evaluating hormones with diurnal cycles such as estradiol, cortisol and melatonin. Further validation studies of the dried urine method should include 24-h correlation and serum correlation for additional hormones or hormone metabolites.

## Conclusions

Our data show that the dried urine assay is a good surrogate for testing reproductive hormones in premenopausal and postmenopausal women. Further, the 4-spot collection method accurately represents results from a full 24-h urine collection. The 4-spot collection methodology is easy for patients and allows for determination of diurnal patterns as well as total 24-h hormone production. Extracted urine from dried samples provides comparable results to liquid urine, and the samples remain stable without refrigeration for at least 12 weeks.

## Additional files

**Additional file 1.** Hormone profiles of serum progesterone versus urinary 5α-pregnanediol (**a**) and serum estradiol and urinary estrone (**b**) in four premenopausal women. Cr, creatinine; αPg, α-pregnanediol.

**Additional file 2.** Interclass correlations of 24-h urine collections for α-pregnanediol (**a**) and estrone (**b**) versus the 4-spot assay. Correlation coefficients reported are Spearman correlations. Cr, creatinine; αPg, α-pregnanediol.

**Additional file 3.** Interclass correlations of dried versus liquid urine for α-pregnanediol (**a**), and estrone (**b**). Correlation coefficients reported are Spearman correlations. Cr, creatinine; αPg, α-pregnanediol.

## Abbreviations

αPg: 5α-pregnanediol
βPg: 5β-pregnanediol
Cr: creatinine
DUTCH: Dried Urine Testing for Comprehensive Hormones
E_2_: estradiol
E_1_: estrone
GC–MS/MS: gas chromatography with tandem mass spectrometry
HRT: hormone replacement therapy
ICC: intraclass correlation coefficients
IQR: interquartile range
RIA: radioimmunoassays

## References

[1] Munro CJ, Stabenfeldt GH, Cragun JR, Addiego LA, Overstreet JW, Lasley BL (1991). Relationship of serum estradiol and progesterone concentrations to the excretion profiles of their major urinary metabolites as measured by enzyme immunoassay and radioimmunoassay. Clin Chem.

[2] Roos J, Johnson S, Weddell S, Godehardt E, Schiffner J, Freundl G (2015). Monitoring the menstrual cycle: comparison of urinary and serum reproductive hormones referenced to true ovulation. Eur J Contracept Reprod Health Care..

[3] Direito A, Bailly S, Mariani A, Ecochard R (2013). Relationships between the luteinizing hormone surge and other characteristics of the menstrual cycle in normally ovulating women. Fertil Steril.

[4] Guthrie R, Susi A (1963). A simple phenylalanine method for detecting phenylketonuria in large populations of newborn infants. Pediatrics.

[5] Waller K, Swan SH, Windham GC, Fenster L, Elkin EP, Lasley BL (1998). Use of urine biomarkers to evaluate menstrual function in healthy premenopausal women. Am J Epidemiol.

[6] Stanczyk FZ, Clarke NJ (2010). Advantages and challenges of mass spectrometry assays for steroid hormones. J Steroid Biochem Mol Biol.

[7] Shackleton C (2010). Clinical steroid mass spectrometry: a 45-year history culminating in HPLC-MS/MS becoming an essential tool for patient diagnosis. J Steroid Biochem Mol Biol.

[8] Krone N, Hughes BA, Lavery GG, Stewart PM, Arlt W, Shackleton CH (2010). Gas chromatography/mass spectrometry (GC/MS) remains a pre-eminent discovery tool in clinical steroid investigations even in the era of fast liquid chromatography tandem mass spectrometry (LC/MS/MS). J Steroid Biochem Mol Biol.

[9] Goebelsmann UBG, Gale JA, Kletzky OA, Nakamura RM, Coulson AH, Lepow IHCRE (1979). Korelitz JJ Serum gonadotropin, testosterone, estradiol and estrone levels prior to and following bilateral vasectomy. Vasectomy: Immunologic and pathophysiologic effects in animals and man.

[10] Probst-Hensch NM, Ingles SA, Diep AT, Haile RW, Stanczyk FZ, Kolonel LN (1999). Aromatase and breast cancer susceptibility. Endocr Relat Cancer.

[11] Bedard M, Martin NJ, Krueger P, Brazil K (2000). Assessing reproducibility of data obtained with instruments based on continuous measurements. Exp Aging Res.

[12] Landis JR, Koch GG (1977). The measurement of observer agreement for categorical data. Biometrics.

[13] Filicori M, Butler JP, Crowley WF (1984). Neuroendocrine regulation of the corpus luteum in the human. Evidence for pulsatile progesterone secretion. J Clin Invest..

[14] Sten T, Bichlmaier I, Kuuranne T, Leinonen A, Yli-Kauhaluoma J, Finel M (2009). UDP-glucuronosyltransferases (UGTs) 2B7 and UGT2B17 display converse specificity in testosterone and epitestosterone glucuronidation, whereas UGT2A1 conjugates both androgens similarly. Drug Metab Dispos.

