# Supplementary figures and images for: Evaluating urinary estrogen and progesterone metabolites using dried filter paper samples and gas chromatography with tandem mass spectrometry (GC–MS/MS)
Source: BMC Chem. 2019 Feb 4;13(1):20. doi: 10.1186/s13065-019-0539-1 (PMC6661742; doi:10.1186/s13065-019-0539-1)

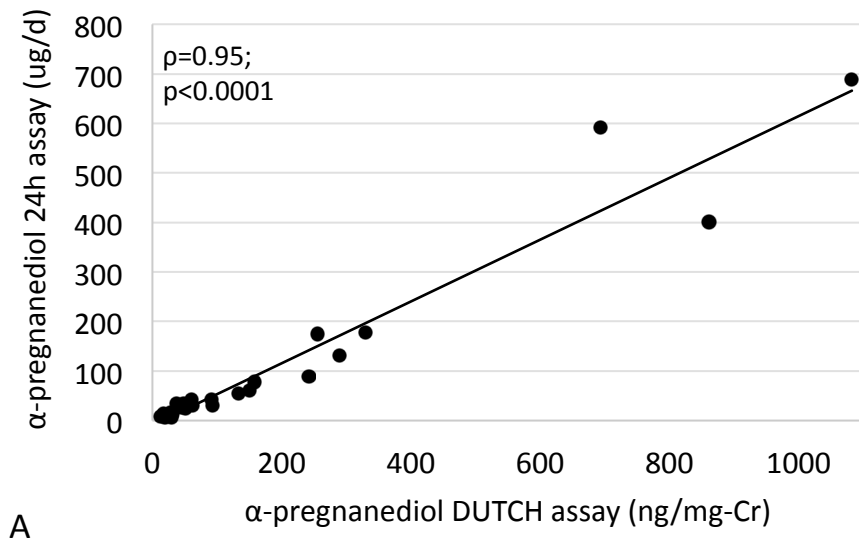

A

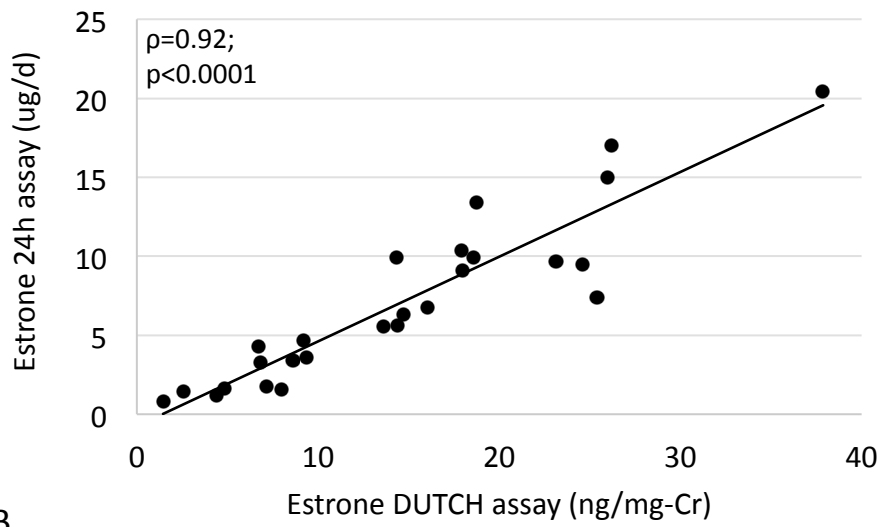

B

Supplement: Supplementary file 2 — Additional file 2. Interclass correlations of 24-h urine collections for α-pregnanediol (a) and estrone (b) versus the 4-spot assay. Correlation coefficients reported are Spearman correlations. Cr, creatinine; αPg, α-pregnanediol. [file 13065_2019_539_MOESM2_ESM.pdf]

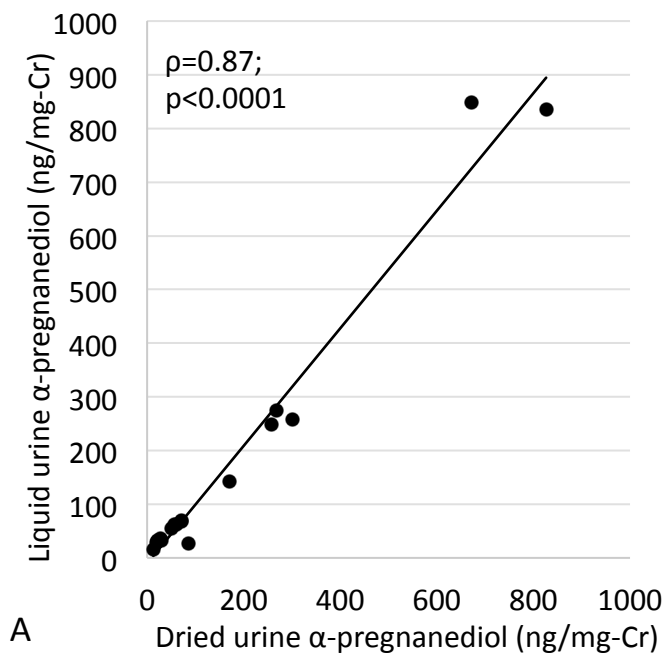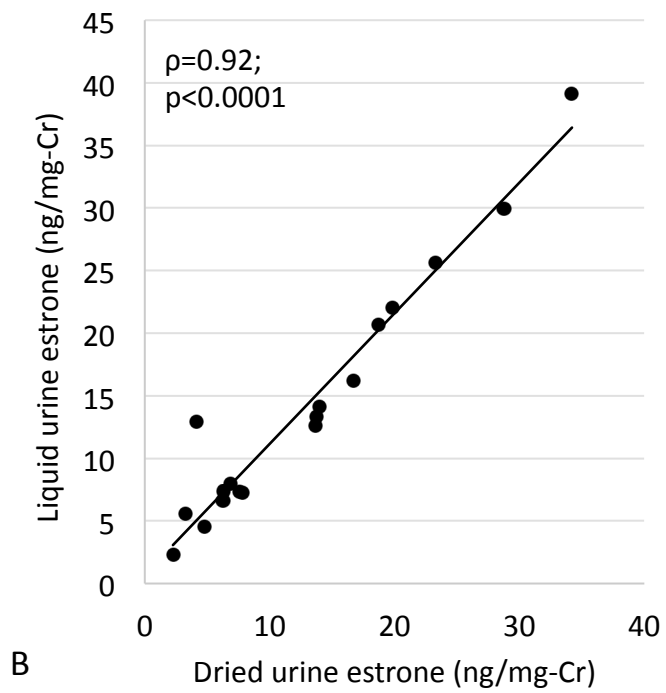

Supplement: Supplementary file 3 — Additional file 3. Interclass correlations of dried versus liquid urine for α-pregnanediol (a), and estrone (b). Correlation coefficients reported are Spearman correlations. Cr, creatinine; αPg, α-pregnanediol. [file 13065_2019_539_MOESM3_ESM.pdf]
