# Supplementary material for: Evaluating urinary estrogen and progesterone metabolites using dried filter paper samples and gas chromatography with tandem mass spectrometry (GC–MS/MS)
Source: BMC Chem. 2019 Feb 4;13(1):20. doi: 10.1186/s13065-019-0539-1 (PMC6661742; doi:10.1186/s13065-019-0539-1)
Supplement: Supplementary file 2 [file 13065_2019_539_MOESM1_ESM.docx]

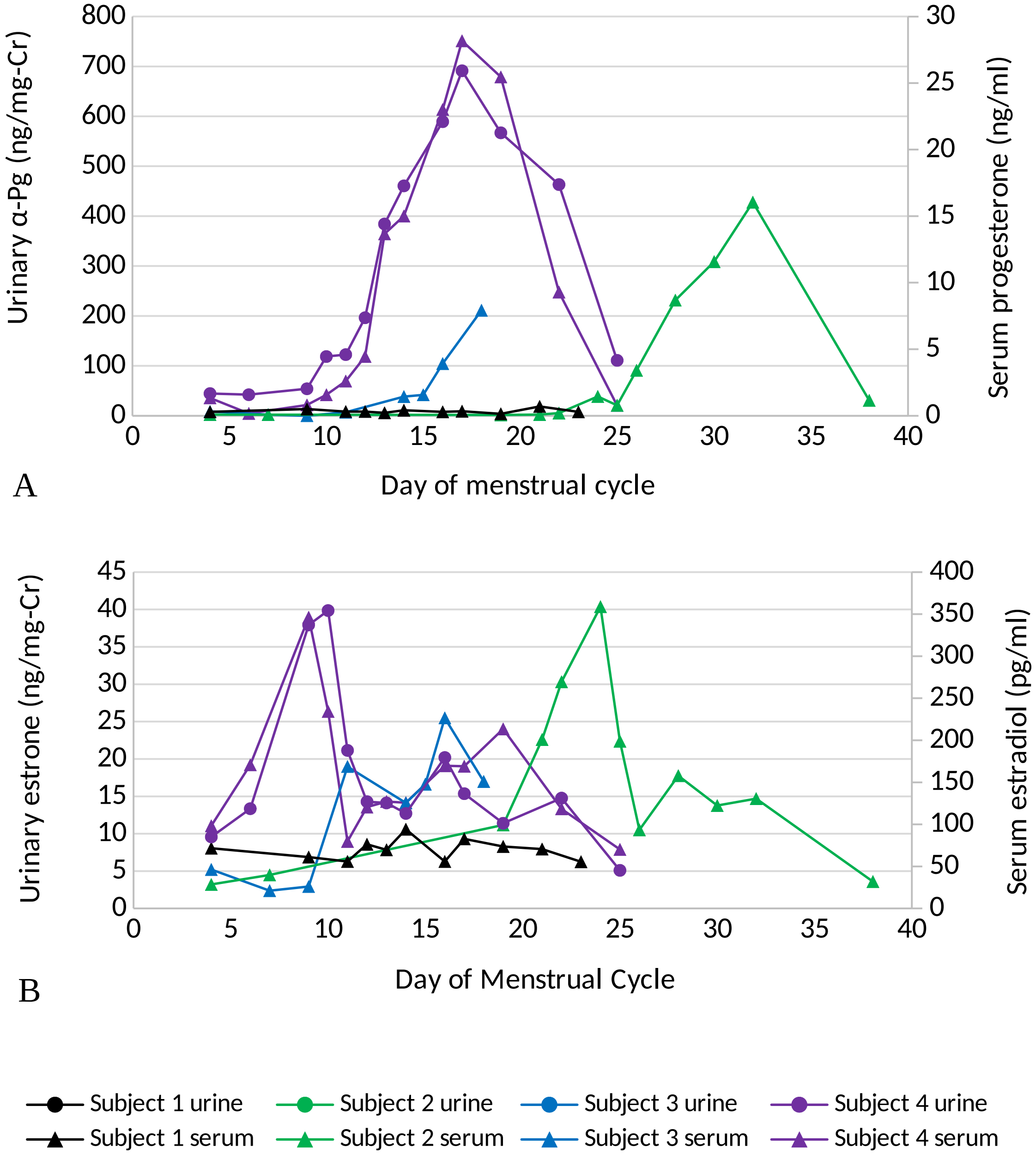
800
700
600
500
400
300
200
100
0
Urinary α-Pg (ng/mg-Cr)
30
25
20
15
10
5
0
Serum progesterone (ng/ml)
0 5 10 15 20 25 30 35 40
A
Day of menstrual cycle
45
40
35
30
25
20
15
10
5
0
Urinary estrone (ng/mg-Cr)
400
350
300
250
200
150
100
50
0
Serum estradiol (pg/ml)
0 5 10 15 20 25 30 35 40
Day of Menstrual Cycle
B
Subject 1 urine
Subject 2 urine
Subject 3 urine
Subject 4 urine
Subject 1 serum
Subject 2 serum
Subject 3 serum
Subject 4 serum
